# Somatic hypermutation of immunoglobulin genes: lessons from proliferating cell nuclear antigen^K164R^ mutant mice

**DOI:** 10.1098/rstb.2008.0223

**Published:** 2008-11-13

**Authors:** Petra Langerak, Peter H.L. Krijger, Marinus R. Heideman, Paul C.M. van den Berk, Heinz Jacobs

**Affiliations:** The Netherlands Cancer InstitutePlesmanlaan 121, 1066 CX Amsterdam, The Netherlands

**Keywords:** proliferating cell nuclear antigen ubiquitylation, somatic hypermutation of Ig genes, mismatch recognition, translesion synthesis, damage tolerance

## Abstract

Proliferating cell nuclear antigen (PCNA) encircles DNA as a ring-shaped homotrimer and, by tethering DNA polymerases to their template, PCNA serves as a critical replication factor. In contrast to high-fidelity DNA polymerases, the activation of low-fidelity translesion synthesis (TLS) DNA polymerases seems to require damage-inducible monoubiquitylation (Ub) of PCNA at lysine residue 164 (PCNA-Ub). TLS polymerases can tolerate DNA damage, i.e. they can replicate across DNA lesions. The lack of proofreading activity, however, renders TLS highly mutagenic. The advantage is that B cells use mutagenic TLS to introduce somatic mutations in immunoglobulin (Ig) genes to generate high-affinity antibodies. Given the critical role of PCNA-Ub in activating TLS and the role of TLS in establishing somatic mutations in immunoglobulin genes, we analysed the mutation spectrum of somatically mutated immunoglobulin genes in B cells from PCNA^K164R^ knock-in mice. A 10-fold reduction in A/T mutations is associated with a compensatory increase in G/C mutations—a phenotype similar to Polη and mismatch repair-deficient B cells. Mismatch recognition, PCNA-Ub and Polη probably act within one pathway to establish the majority of mutations at template A/T. Equally relevant, the G/C mutator(s) seems largely independent of PCNA^K164^ modification.

## 1. Introduction

To establish an effective immune response against innumerable infectious agents, B cells are capable of generating highly specific antibodies. The primary antibody repertoire is shaped by site-specific recombination of immunoglobulin (Ig) variable (V), diversity (D) and joining (J) gene segments of the Ig loci in a RAG-dependent manner (Jung *et al*. 2006). As V(D)J recombination is restricted to the CDR3 region only, primary antibodies are generally of low affinity. To improve affinity, B cells have a unique capacity to undergo a second round of diversification involving the entire variable region, a process known as somatic hypermutation (SHM; Di Noia & Neuberger 2007). During this process, point mutations are introduced into the variable region of Ig genes at an astonishing rate of approximately 10^−3^ base pairs per generation, six orders of magnitude greater than spontaneous mutagenesis (Rajewsky 1996). Despite extensive knowledge of the physiology, timing, sequence preference and mutation spectrum of SHM, the molecular mechanism remained elusive for decades.

The breakthrough was provided by the discovery of activation-induced cytidine deaminase (AID; Muramatsu *et al*. 1999), a member of the *Apobec* gene family encoding zinc-dependent cytosine deaminases. AID-deficient B cells lack SHM and class switch recombination (CSR; Muramatsu *et al*. 2000; Revy *et al*. 2000; Bross *et al*. 2002). This enzyme is expressed in B cells of the germinal centre, binds single-stranded DNA (ssDNA) and initiates SHM and CSR by intentionally introducing U/G mismatches in the Ig DNA (Chaudhuri & Alt 2004; Di Noia & Neuberger 2007). As high-fidelity replication over uracils only results in C to T and G to A transitions, the deamination of cytosine residues itself cannot explain the spectrum of base changes characteristic of somatically mutated Ig genes. Therefore, mutagenic processing at and around the initial uracil lesion must take place.

Observations in mice deficient in mismatch repair (MMR) and base excision repair (BER) pathways revealed alternative pathways on how these mutations are generated. While B cells deficient for the mismatch recognition proteins MutS homologue 2 (MSH2) and MSH6 exhibited a strong reduction in mutations at A/T base pairs, B cells lacking the BER protein uracil DNA glycosylase 2 (UNG2) showed a marked shift in the mutations generated at G/C base pairs from transversions to transitions, leaving the mutations at A/T base pairs unaffected (Rada *et al*. 1998, 2002; Wilson *et al*. 2005). As these repair pathways are normally error-free, and highly effective in dealing with U/G lesions, the following question arises: what makes these pathways so mutagenic?

## 2. Role of translesion synthesis in SHM

Over the past two decades, novel DNA polymerases have been identified and characterized. These polymerases lack proofreading activity and share the unique capacity to bypass DNA lesions, i.e. continue replication in the presence of genotoxic lesions and thereby tolerate the damage. These polymerases are referred to as translesion synthesis (TLS) polymerases and include polymerase η (Polη), Polι, Polκ, reversionless 1 (Rev1) of the Y-family of DNA polymerases, Polζ of the B-family (Prakash *et al*. 2005) and Polθ of the A-family (Seki *et al*. 2004). TLS proceeds in a two-step mode: (i) incorporation of a nucleotide directly opposite to the lesion, and (ii) elongation from the distorted or bulky non-Watson–Crick base pairs. Polη, a polymerase that is absent in patients with the variant form of xeroderma pigmentosum (XPV; Cordonnier & Fuchs 1999; Johnson *et al*. 1999), was first linked to SHM. B cells from these patients showed an altered spectrum of somatic point mutations. A significant reduction in mutations at A/T base pairs was associated with an increased mutation frequency at template G/C (Di Noia & Neuberger 2007). In addition, knockdown and antisense RNA targeting of Polζ, a polymerase known to efficiently extend from mispaired primers, resulted in impaired SHM at all base pairs, in both human and murine B cells (Diaz *et al*. 2001; Zan *et al*. 2001). These observations provided the first evidence for the involvement of TLS polymerases in establishing defined point mutations in hypermutated Ig genes and stimulated efforts in identifying other TLS polymerases involved in this process.

## 3. Rev1 generates C/G to G/C transversions

The TLS polymerase Rev1 protein was initially identified in a genetic screen for UV-induced reversions in yeast (Lemontt 1971). Rev1 is selective in its nucleotide incorporation activity as it only incorporates dCMP, and therefore in its strictest sense should be regarded as a deoxycytidyl transferase rather than a bona fide DNA polymerase. *In vitro*, it is capable of bypassing both uracil residues and abasic sites, two natural intermediates for SHM (Nelson *et al*. 1996). Orthologues of Rev1 were identified in *Saccharomyces cerevisiae*, *Caenorhabditis elegans*, chicken, mouse and man. In addition to the five conserved motifs characteristic of the catalytic domain of Y-family polymerases, Rev1 harbours a BRCA1 C-terminal (BRCT) domain in its N-terminus (Gerlach *et al*. 1999). This domain binds proteins that are phosphorylated by DNA damage-activated protein kinases ataxia telangiectasia related (ATR) and ataxia telangiectasia mutated (ATM) (Manke *et al*. 2003; Rodriguez *et al*. 2003). The BRCT domain of Rev1 was shown to regulate TLS of abasic sites in yeast (Haracska *et al*. 2001). However, together with our collaborators, we showed that hypermutated Ig genes of memory B cells derived from Rev1 mutant mice lacking the N-terminal BRCT domain revealed no changes in the base-exchange pattern. This indicates that the BRCT domain is dispensable in establishing somatic mutations in the V regions of Ig genes (Jansen *et al*. 2005), leaving the possibility that the catalytic domain of Rev1 might play a role in SHM. Indeed, we showed that memory B cells derived from Rev1-deficient mice exhibited an altered base-exchange pattern (Jansen *et al*. 2006). In agreement with the reported *in vitro* ability of Rev1 to bypass uracils and abasic sites by incorporating cytosine residues opposite to these lesions, C to G and G to C transversions were significantly reduced in the absence of Rev1. This reduction was associated with an increase in A to T, C to A and T to C mutations. In the presence of Rev1, an abasic site—derived from cytosine deamination by AID followed by the removal of the uracil by UNG2 during SHM—will be bypassed by the incorporation of a cytidine residue, leading to C to G and G to C transversions. In the absence of Rev1, however, other TLS polymerases with a distinct mutation signature are likely to bypass this lesion, thereby favouring the introduction of other mutations. Compensatory activation of Polη (mainly responsible for A and T mutations) and presumably Polι may be responsible for the observed increase in other mutations. Interestingly, *in vitro* studies with Polι have shown a preference for this polymerase to insert either G or T residues opposite to an abasic site (Zhang *et al*. 2001). While incorporation of G opposite to an abasic site will faithfully restore the initial lesion, the introduction of T would result in C to A transversions. In addition, at template T, Polι prefers to make T/G mispairs rather than T/A base pairs (Zhang *et al*. 2000), which could also explain the increase in T to C transitions in the absence of Rev1. No changes in SHM were, however, observed in B cells derived from a *129/J* mouse strain that carry a spontaneous nonsense mutation in the Polι gene (McDonald *et al*. 2003). Western blot analysis on testis extracts indeed showed the absence of Polι in this strain. Nevertheless, it has been described that there may be tissue-specific and functional alternative splice forms of Polι, and Polι activity seems to be retained in the brain extract from this mouse strain (Gening *et al*. 2006). In this context, *129/J*-derived B cells should be tested for the presence of hypomorph versions of Polι. Therefore, one cannot exclude that Polι is responsible for some mutations increased in the absence of Rev1. Analysis of B cells derived from a mouse strain carrying a targeted deletion of Polι will solve this issue.

## 4. Differential preference in recruiting TLS polymerases during MMR and BER

Intriguingly, the base-exchange spectrum observed in mutated Ig genes of Polη-deficient B cells is similar to the pattern in MSH2-deficient cells (Jacobs *et al*. 1998; Rada *et al*. 1998; Wiesendanger *et al*. 2000). Both spectra revealed a strong reduction in mutations at template A and T, while G and C mutations are relatively unaffected. In addition, C to G and G to C transversions that are generated by Rev1 are strongly reduced in UNG2-deficient cells (Rada *et al*. 2002). These data suggest that MMR and BER make use of distinct mutagenic DNA polymerases to generate specific point mutations. The deamination of cytosine residues by AID generates U/G mispairs. These U/G mispairs are recognized by MutSα, a complex of MSH2 and MSH6 (Wilson *et al*. 2005). While MMR normally requires the recruitment and activation of the MutL complex, which makes the DNA accessible for exonucleases, MutL is dispensable for SHM (Li & Modrich 1995; Erdeniz *et al*. 2007). The exonuclease removes a single-stranded stretch of the DNA containing the uracil residue (Genschel *et al*. 2002). Polymerases can restore the DNA using the remaining ssDNA as a template for resynthesis. While high-fidelity polymerases normally fill in the single-stranded gap, TLS polymerase Polη appears to be operative during SHM.

Alternatively, the uracil residue can be recognized and cleaved by the BER enzyme UNG2, leaving behind an abasic site (Tomilin & Aprelikova 1989). The remaining sugar-phosphate backbone is nicked by an AP endonuclease (APE) to allow high-fidelity polymerase beta (Polβ) to remove the sugar-phosphate of the abasic site and simultaneously incorporate the missing nucleotide and restore the damage accurately (Dianov *et al*. 1992; Matsumoto & Kim 1995). In addition, ‘long-patch’ BER can be used. APEs introduce a nick 5′ of the lesion (Matsumoto *et al*. 1994; Klungland & Lindahl 1997). High-fidelity polymerases will synthesize a stretch of several nucleotides while displacing the old strand (Klungland & Lindahl 1997; Stucki *et al*. 1998). The resulting flap, a stretch of ssDNA, is then removed by flap endonuclease 1 (FEN1; Klungland & Lindahl 1997; Kim *et al*. 1998). During SHM, however, TLS polymerases are recruited to perform error-prone resynthesis. In line with observations that Rev1 is able to bypass abasic sites *in vitro* and *in vivo* (Nelson *et al*. 1996), it appears that Rev1 is at least partially responsible for the bypass of UNG2-generated abasic sites during SHM. This raises a new question: what regulates the recruitment of low-fidelity TLS polymerases rather than high-fidelity replicative polymerases in resolving AID-induced lesions?

## 5. Power of the rings: proliferating cell nuclear antigen

**Figure 1:**
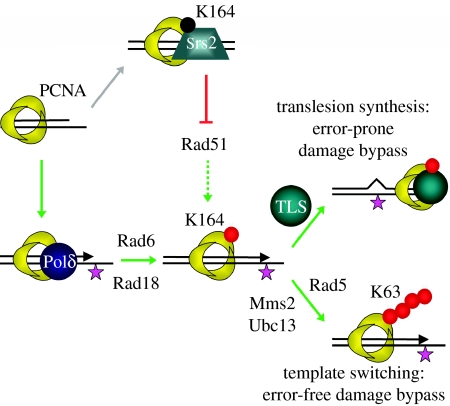
Role of the Rad6 epistasis group in DNA damage bypass. The ring-shaped PCNA homotrimer encircles DNA and, by tethering DNA Polδ to the template, it serves as an important processivity factor for DNA replication. In the presence of DNA damage (indicated by an asterisk), PCNA becomes monoubiquitinated (red circle) at the lysine residue 164 by the ubiquitin conjugating/ligating complex Rad6/Rad18. PCNA-Ub can directly activate TLS polymerases (such as Polη, Rev1 and Polζ), enabling an error-prone damage bypass. Alternatively, K63-linked polyubiquitylation of PCNA-Ub by the Rad5/Mms2/Ubc13 complex enables template switching and thus an error-free damage bypass. Besides ubiquitylation, PCNA can also be SUMOylated (black circle) at the lysine residue 164. PCNA–SUMO recruits the anti-recombinogenic Srs2 helicase, which prohibits Rad51 filament formation and is thought to favour damage tolerance indirectly.

Non-instructive DNA lesions, such as abasic sites, cause problems for high-fidelity polymerases and lead to replication fork stalling. If the ‘stalling’ lesion is not repaired, the replication fork may collapse (Tercero & Diffley 2001). Such a collapse can generate double-strand breaks, which can in turn trigger cell death (McGlynn & Lloyd 2002). To prevent such fatal lesions during replication, eukaryotic cells seem to be equipped with two alternative DNA damage tolerance pathways: template switching and TLS, which can be activated during the S-phase of the cell cycle (Murli & Walker 1993; Lawrence 1994; Haracska *et al*. 2001; Friedberg 2005). Damage tolerance allows a cell to continue DNA synthesis without an *a priori* repair of the initial lesion. Template switching uses intact DNA of the sister chromatid as a template to continue replication and is therefore error-free (Zhang & Lawrence 2005). While template switching bypasses the lesion indirectly, TLS enables replication to continue directly on the damaged template. However, depending on the type of damage and the nature of the TLS polymerase involved, TLS can be highly error-prone (Friedberg *et al*. 2002; Prakash *et al*. 2005). While damage tolerance probably evolved to increase the fitness of a cell in response to specific DNA damage, as mentioned previously, B cells take advantage of this error-prone system in order to introduce mutations in the variable region of the Ig locus and eventually improve the affinity of their antibodies for the cognate antigen. This raises three central questions: what determines the decision making between conventional repair and damage tolerance; how are low-fidelity polymerases activated; and how does the system decide between error-prone TLS and error-free template switching? Studies in *S. cerevisiae* have shed light on the mechanism underlying the selective (in)activation of these critical pathways. Both modes of lesion bypass appear to be controlled by specific post-translational modifications of the homotrimeric DNA sliding clamp proliferating cell nuclear antigen (PCNA; Hoege *et al*. 2002; figure 1). PCNA tethers DNA polymerases to their substrate and thereby serves as a critical processivity factor for DNA synthesis. The use of PCNA as a sliding clamp for TLS polymerases during damage bypass implies a polymerase switch from the high-fidelity Polδ to low-fidelity TLS polymerases (Plosky & Woodgate 2004). During replication, Polδ binds PCNA through its PIP (PCNA-interacting peptide) box, characterized by the consensus motif QXX(M/L/I)XX (F/Y)(F/Y) (Warbrick 1998). At this stage, TLS polymerases associate weakly with PCNA. When the high-fidelity replication machinery is stalled upon encountering a lesion, PCNA becomes monoubiquitylated at its lysine residue 164 (PCNA^K164^; Hoege *et al*. 2002). At that moment, TLS polymerases are recruited to the monoubiquitylated PCNA (PCNA-Ub) through the combined affinity of the PIP box and ubiquitin-binding domains, i.e. a Ub-binding motif (UBM) or a Ub-binding zinc finger (UBZ) resulting in a transient displacement of the high-fidelity polymerase Polδ (Bienko *et al*. 2005). The ubiquitin-conjugating/ligating complex Rad6/Rad18 (E2/E3) mediates the monoubiquitylation of PCNA and thereby is thought to enable polymerase switching and activation of TLS-dependent damage tolerance. The alternative pathway of damage tolerance, i.e. template switching, requires further polyubiquitylation of the monoubiquitylated PCNA (Hoege *et al*. 2002). The heterodimeric E2 ubiquitin-conjugating complex consisting of Ubc13 and Mms2 cooperates with the RING-finger E3 Rad5 to form a ubiquitin conjugase/ligase complex that enables specific lysine 63-linked polyubiquitylation (Torres-Ramos *et al*. 2002). How polyubiquitylated PCNA mechanistically activates the error-free branch of damage tolerance and the relevance of this pathway in mammals remains to be elucidated.

The observations that TLS polymerases are required during SHM to introduce mutations in the variable region of Ig genes and the fact that the RAD6 epistasis group has functional orthologues in higher eukaryotes suggested that this pathway is of general importance. In support of this notion, UV irradiation of both human and murine cells was shown to lead to the monoubiquitylation of PCNA at the conserved K164 residue, which resulted in the accumulation of TLS polymerases at sites of DNA damage (Kannouche *et al*. 2004). This implies a conserved mechanism between yeast and mammals in the recruitment and activation of TLS polymerases. To test whether this mode of polymerase activation and inactivation is operative in mammalian cells and related to the generation of somatic mutations in hypermutating B cells, several approaches were considered. One strategy to abolish PCNA ubiquitylation is to remove the ubiquitin-conjugating enzyme Rad6. The inactivation of a ubiquitin-conjugating enzyme (E2) can, however, be problematic, as it is the ubiquitin-ligating enzyme (E3) that determines the substrate specificity, and one E2 can collaborate with multiple E3s. Indeed, Rad6 was shown to interact with more than one E3, i.e. Rad18 and Ubr1 (for protein degradation via the N-end rule; Dohmen *et al*. 1991; Bailly *et al*. 1997; Xin *et al*. 2000). In addition, there are two homologues of Rad6 in mice, mHR6A and mHR6B, further complicating the analysis of Rad6-deficient mice (Koken *et al*. 1992). Nevertheless, knockout mice for both of these proteins have been generated. While single knockout mice are viable and still capable of damage bypass, double knockout mice are not born (Roest *et al*. 2004). Other options are to remove the E3 Rad18 or alter the modified amino acid of the PCNA molecule. We decided to keep the mutation as subtle as possible and generated PCNA mutant mice that contain a lysine residue 164 to arginine mutation (PCNA^K164R^). This subtle point mutation allows the specific analysis of PCNA^K164^ modification without removing complete proteins or interfering with other functions of this pivotal protein.

## 6. Generation, survival and development of PCNA^K164R^ knock-in mice

The targeting construct for the PCNA^K164R^ knock-in mutation was generated as described previously (Langerak *et al*. 2005). For the generation of PCNA^K164R^ mutant mice, embryonic stem (ES) cells were electroporated to deliver the targeting construct (Capecchi 1989). As homologous recombination is quite inefficient in murine ES cells—non-homologous integrations usually outnumber homologous integrations—we established an adapted multiplex ligation-dependent probe amplification method that can quantify, identify and distinguish homologous and non-homologous recombination events in one reaction (Langerak *et al*. 2005). Applying this screening method, ES cell clones carrying a homologous recombined PCNA^K164R^ allele were selected to introduce the mutation in the mouse germ line. To test whether homozygous carriers of the PCNA^K164R^ mutation are viable, the offspring (*n*=397) from 70 intercrosses between heterozygous parents were genotyped. Surprisingly, not only heterozygous but also homozygous PCNA^K164R^ mutant mice were born, albeit at a sub-Mendelian frequency (Langerak *et al*. 2007), i.e. 5 instead of the expected 25 per cent. Apparently, the PCNA^K164R^ mutation is compatible with mammalian survival. However, these mice still contained the PGK-puroΔtk selection cassette expressing a puromycin-resistant gene fused to a truncated thymidine kinase gene under the PGK promoter. To exclude a lethal gene dosage effect of the PGK-puroΔtk cassette in homozygous embryos, we generated PCNA^K164R^flpe mice, in which the selection cassette was deleted by crossing to the Flp-deleter strain (Rodriguez *et al*. 2000). To our surprise, in the absence of a selection cassette, the frequency of finding homozygous mutants from heterozygous PCNA^K164R^ flpe parents was greatly improved; that is, from 125 pups, 39/31 expected were wild-type, 66/63 expected were heterozygous and, most importantly, 20/31 were homozygous (16% observed versus 25% expected). Apparently, the PCNA^K164R^ mutation is less detrimental than concluded previously (Langerak *et al*. 2007). These observations emphasize the importance of a deletable selection cassette in gene targeting. Surviving homozygous PCNA^K164R^ and PCNA^K164R^ flpe mutant mice develop and grow normally, but are infertile. The infertility is caused by a severe hypotrophy of the gonads, which is associated with a virtually complete lack of germ cells. These observations suggest a selective requirement for wild-type PCNA in germ cell development. In this regard, a possible explanation for the deficiency of germ cell development could be that the K164R mutation prohibits not only monoubiquitylation of PCNA, but also pro-recombinogenic polyubiquitylation, as well as anti-recombinogenic modification with small ubiquitin-like modifier (SUMO; Hoege *et al*. 2002). In yeast, PCNA–SUMO was shown to recruit Srs2, an anti-recombinogenic helicase (Pfander *et al*. 2005). This helicase disrupts Rad51 filament formation, which is required for recombination. Srs2 is upregulated during meiosis at the time coincident with the commitment to recombination in yeast and is required for the actual repair of double-strand breaks (Pfander *et al*. 2005; Houston & Broach 2006). Although PCNA–SUMO has not been detected in mammalian cells yet, these observations suggest a possible role for PCNA–SUMO and Srs2 during meiosis, to regulate the repair of double-strand breaks that are required to complete crossovers. In PCNA^K164R^ mutant mice, Srs2 recruitment via PCNA–SUMO cannot take place. It is therefore possible that double-strand breaks are induced that can trigger damage-induced cell death of germ cells.

## 7. Somatic hypermutation in PCNA^K164R^ mutant B cells

**Figure 2:**
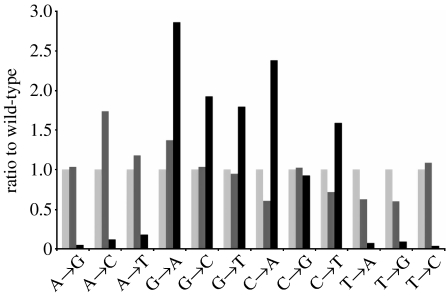
SHM is altered in the absence of PCNA^K164^ modification. Mutations found in memory B cells isolated from five individual mice of each genotype (light grey bars, PCNA^+/+^; dark grey bars, PCNA^+/K164R^; black bars, PCNA^K164R/K164R^) were pooled. The average mutation load (number of point mutations in the 5′ region of the J_H_4 intron) was 5.8, 5.6 and 4.5 per cell in wild-type, heterozygous and homozygous PCNA^K164R^ mutants. The overall distribution of the mutations was found to be similar between wild-type, hetero- and homozygous PCNA^K164R^ mice. However, the mutation spectrum was significantly altered. Point mutations from wild-type (*n*=502), heterozygous (*n*=255) and homozygous (*n*=503) are analysed (Langerak *et al*. 2007). To simplify the comparison of the mutation spectra, the base change patterns were normalized to wild-type. The percentage of each base change in the wild-type is set to 1 and the relative ratio of mutant to wild-type is shown. No major differences were observed between wild-type and heterozygous PCNA^K164R^ mice. In homozygous mice, A/T mutations are virtually lacking. The decrease in generating mutations at template A/T is associated with a relative and absolute increase in G/C mutations, with the notable exception of C to G transversions. The lack of PCNA ubiquitylation in homozygous mutants causes a strong increase in G to A and C to T transitions. With the exception of C to G transversions, all other exchanges found in homozygous mutant B cells differ significantly as determined by the chi-squared test (*p*-value<0.05).

Mutagenic processing of AID-induced lesions involves low-fidelity polymerases, i.e. TLS polymerases, and thus the activation of the error-prone DNA damage tolerance pathway, to establish somatic mutations in hypermutating B cells. Analysis of the SHM pattern of B cells derived from homozygous PCNA^K164R^ mice therefore allowed us to determine whether the modification of PCNA^K164^ is involved in the generation of mutations during SHM. Interestingly, as described previously (Langerak *et al*. 2007), PCNA^K164R^ B cells are able to mutate their Ig genes at a mutation frequency of 0.79 per cent when compared with 1.06 and 1.10 per cent for wild-type and heterozygous mutants, respectively (Langerak *et al*. 2007). Considerable changes in the base-exchange pattern were found in homozygous but not in heterozygous PCNA^K164R^ B cells (figure 2). A 90 per cent decrease in mutations at template A/T was observed, clearly revealing the existence of a major PCNA-dependent A/T mutator pathway in SHM. Since most A/T mutations—normally accounting for half of all mutations—are lacking in homozygous PCNA^K164R^ B cells, the mutation frequency is expected to be reduced by 50 per cent. The finding that the overall mutation frequency is reduced by only 25 per cent suggests a partial compensation by G/C-mutator activities, with the noted exception of C to G transversions. As reported previously, Rev1 is involved in the generation of both C to G and G to C transversions in mutated Ig genes (Jansen *et al*. 2006). Remarkably, both TLS polymerases Polη and Rev1 were shown to depend on binding to monoubiquitylated PCNA for an effective damage bypass (Guo *et al*. 2006*a*,*b*; Parker *et al*. 2007). The relative reduction in C to G transversions observed in PCNA mutant B cells may therefore be explained by an impaired Rev1 activity.

Interestingly, not only PCNA^K164R^ mutant B cells showed a strong reduction in A/T mutagenesis; a very similar phenotype was observed in B cells from Polη^−/−^ and MMR-deficient MSH2^−/−^ or MSH6^−/−^ mice (Jacobs *et al*. 1998; Rada *et al*. 1998; Zeng *et al*. 2001; Delbos *et al*. 2005). Except for Polη-deficient mice, which have a slightly higher remaining frequency of A/T mutations, both the frequency of A/T mutations and the base-exchange pattern are very similar between the different mutants. This suggests that mismatch recognition, monoubiquitylation, PCNA-Ub and Polη act within the same pathway to establish most A/T mutations during SHM. In support of this notion, we found that Polη foci formation in UV-irradiated primary homozygous PCNA^K164R^ mouse embryo fibroblasts is severely reduced (Langerak *et al*. 2007, unpublished data). These data indicate that Polη lies downstream of PCNA-Ub. In order to obtain genetic evidence that mismatch recognition, PCNA^K164^ modification and Polη are epistatic in generating A/T mutations, intercrosses between PCNA^K164R^ and Polη^−/−^, and PCNA^K164R^ and MSH2^−/−^ mice are currently analysed in our laboratory.

Considering the trimeric nature of the PCNA sliding clamp, it is interesting that the mutation frequency, mutation load and base-exchange pattern in heterozygous PCNA^K614R^ mice do not differ considerably from wild-type. These observations have implications regarding the dependence of Polη activity on the ubiquitylation status of the PCNA trimer. Analysis of PCNA mRNA of heterozygous B- and T-cell blasts revealed that both PCNA alleles are transcribed at equal levels (Langerak *et al*. 2007). Therefore, in this setting, only one out of eight PCNA trimers will have a 3K composition and become ubiquitylated at all three K164 residues. Although normally all monomers within the PCNA trimer can become ubiquitylated (Kannouche *et al*. 2004; Haracska *et al*. 2006), the unimpaired generation of A/T mutations in heterozygous PCNA^K164R^ B cells favours the idea that a single PCNA-Ub within the PCNA trimer suffices in activating Polη.

In conclusion, SHM is a process that is initiated by the deamination of cytosine residues in the variable region of the Ig locus by AID. The MMR and BER pathways present alternative routes for the generation of somatic mutations. Although these pathways normally recruit faithful high-fidelity polymerases to repair the DNA damage, during SHM, low-fidelity polymerases are employed. The A/T mutator Polη, which belongs to the MMR-dependent arm of SHM, appears to depend on PCNA^K164^ monoubiquitylation for its activation. As Rev1 contains a ubiquitin-binding domain, similar to Polη, the role for PCNA ubiquitylation to allow Rev1 to perform its dCMP transferase needs to be established. However, most G/C mutations might actually depend on the ubiquitylation of an alternative ring, the heterotrimeric Rad9/Rad1/Hus1 sliding clamp or 9-1-1 ring (see below).

It has been described that all Y-family polymerases contain ubiquitin-binding motifs and depend on monoubiquitylated PCNA to be recruited to the sites of damage to perform their function. The described results on the role of PCNA modification in SHM imply that Polη indeed requires PCNA-Ub, and suggest that this is also true for Rev1. However, to determine the absolute dependence of TLS polymerases on PCNA for functionality, intercrosses between PCNA^K164R^ mutant mice and mice deficient for each of the Y-family polymerases are required. It is striking that G/C mutations are not impaired in the absence of PCNA^K164^ modification. While Polδ activity can easily explain the increased frequency of G to A and C to T transitions, transversions at G and C probably relate to an UNG2-dependent G/C mutator activity that allows other TLS polymerases (mainly G/C mutators) to become activated, indicating that not all TLS polymerases depend on PCNA modification but yet may depend on the 9-1-1 ring.

## 8. Power of the rings: the 9-1-1 ring a missing link

The heterotrimeric DNA sliding clamp Rad9/Rad1/Hus1 complex (also known as the 9-1-1 complex) is structurally very similar to PCNA (Griffith *et al*. 2002). It was shown in yeast to interact with TLS polymerases and induce TLS independent of PCNA^K164^ modification (Kai & Wang 2003; Sabbioneda *et al*. 2005; Chen *et al*. 2006). As described recently, the Rad9 clamp protein is phosphorylated by Rad3, on Thr 225, within the PCNA-like domain (Kai *et al*. 2007). While a Rad9 (T225C) mutant induces highly active TLS, phosphorylation of this residue prevents inappropriate Rad51-dependent recombination, possibly by redirecting repair to the error-free branch of the Rad6 repair pathway. Most interestingly, the 9-1-1 complex has recently been identified as an alternative substrate of the Rad6/18 ubiquitin conjugase/ligase complex (Fu *et al*. 2008). The 9-1-1 complex may therefore provide a platform for PCNA-independent TLS during SHM, and a closer analysis of this sliding clamp is likely to shed light on the regulation of the G/C mutator activity in somatic hypermutating B cells and other cells.

In summary, monoubiquitylation of PCNA^K164^ and activation of TLS have a dual, mutagenic and anti-mutagenic, physiological purpose. During SHM of Ig genes, Polη is recruited by PCNA-Ub to generate almost all A/T mutations. During replication of UV-damaged genome, Polη is recruited to correctly insert two adenines opposite to the TT dimers, thereby increasing the fitness of eukaryotes in response to genotoxic UV stress. Given the defined mutation signatures of individual TLS polymerases, the analysis of hypermutated Ig genes provides an effective read-out system for the activity of diverse TLS polymerases and their regulation. While ubiquitylation of PCNA serves to generate A/T mutations during error-prone MMR, the next episode on the power of rings in controlling the outcome of SHM is likely to reveal that TLS polymerases involved in G/C mutations do depend on the ubiquitylation of the 9-1-1 ring.
